# Fulminant Viral Hepatitis in Two Siblings with Inherited IL-10RB Deficiency

**DOI:** 10.1007/s10875-022-01376-5

**Published:** 2022-10-29

**Authors:** Cecilia B. Korol, Serkan Belkaya, Fahad Alsohime, Lazaro Lorenzo, Stéphanie Boisson-Dupuis, Joseph Brancale, Anna-Lena Neehus, Silvia Vilarinho, Alsum Zobaida, Rabih Halwani, Saleh Al-Muhsen, Jean-Laurent Casanova, Emmanuelle Jouanguy

**Affiliations:** 1 Laboratory of Human Genetics of Infectious Diseases, Necker Branch, INSERM U1163, Necker Hospital for Sick Children, Paris, France; 2 Imagine Institute, Paris Cité University, Paris, France; 3 St. Giles Laboratory of Human Genetics of Infectious Diseases, Rockefeller Branch, The Rockefeller University, New York, NY USA; 4 Present Address: Department of Molecular Biology and Genetics, Ihan Dogramaci Bilkent University, Ankara, Turkey; 5 Immunology Research Laboratory, College of Medicine, King Saud University, Riyadh, Saudi Arabia; 6 Department of Internal Medicine, Section of Digestive Diseases, and Department of Pathology, Yale University School of Medicine, New Haven, CT USA; 7 Department of Pediatrics, King Saud University Medical City, Riyadh, Saudi Arabia; 8 Department of Clinical Sciences, College of Medicine, Sharjah Institute for Medical Research (SIMR), University of Sharjah, Sharjah, United Arab Emirates; 9 Howard Hughes Medical Institute, New York City, NY USA; 10 Department of Pediatrics, Necker Hospital for Sick Children, Paris, France

**Keywords:** Fulminant viral hepatitis, inherited IL-10RB deficiency, inborn error of immunity, hepatitis A virus, early-onset inflammatory bowel disease, autosomal recessive disease, IL10RB, IL-10, IL-22, IL-26, IFN-λ, IL-18, IL-18BP, excessive IFN-gamma

## Abstract

Fulminant viral hepatitis (FVH) caused by hepatitis A virus (HAV) is a life-threatening disease that typically strikes otherwise healthy individuals. The only known genetic etiology of FVH is inherited IL-18BP deficiency, which unleashes IL-18-dependent lymphocyte cytotoxicity and IFN-γ production. We studied two siblings who died from a combination of early-onset inflammatory bowel disease (EOIBD) and FVH due to HAV. The sibling tested was homozygous for the W100G variant of *IL10RB* previously described in an unrelated patient with EOIBD. We show here that the out-of-frame *IL10RB* variants seen in other EOIBD patients disrupt cellular responses to IL-10, IL-22, IL-26, and IFN-λs in overexpression conditions and in homozygous cells. By contrast, the impact of in-frame disease-causing variants varies between cases. When overexpressed, the W100G variant impairs cellular responses to IL-10, but not to IL-22, IL-26, or IFN-λ1, whereas cells homozygous for W100G do not respond to IL-10, IL-22, IL-26, or IFN-λ1. As IL-10 is a potent antagonist of IFN-γ in phagocytes, these findings suggest that the molecular basis of FVH in patients with IL-18BP or IL-10RB deficiency may involve excessive IFN-γ activity during HAV infections of the liver. Inherited IL-10RB deficiency, and possibly inherited IL-10 and IL-10RA deficiencies, confer a predisposition to FVH, and patients with these deficiencies should be vaccinated against HAV and other liver-tropic viruses.

**Supplementary Information:**

The online version contains supplementary material available at 10.1007/s10875-022-01376-5.

## Introduction

The most common agents of human viral hepatitis are hepatitis A virus (HAV), hepatitis B virus (HBV), hepatitis C virus (HCV), and hepatitis E virus (HEV) [1–3]. HBV and HCV typically cause chronic infections, which can underlie fibrosis, cirrhosis, and hepatocellular carcinoma [4]. By contrast, infections with HAV and HEV are always acute and mostly benign [5]. In rare cases, primary infection with HAV, HBV, or HEV can lead to fulminant viral hepatitis (FVH), which is characterized by life-threatening hepatic failure. FVH due to HCV is exceedingly rare [6, 7]. In the vast majority of cases, FVH strikes otherwise healthy individuals with normal resistance to other infectious agents [8–11]. There are divergent estimates of the incidence of FVH, from ~ 0.6 cases per million individuals in Scotland [12] to ~ 1.6 cases per million individuals in Thailand [13]. The incidence of FVH due to HAV has not been precisely determined but is estimated at about 0.5% in individuals with symptomatic HAV infection [14]. The outcome of FVH is poor, regardless of the causal virus, with less than 25% survival in the absence of liver transplantation [8, 9, 15–17].

FHV is not caused by particular HAV genotypes [18, 19] and is not epidemic. It, therefore, seems unlikely that FVH is caused by more virulent viral isolates. It is typically sporadic, but a few familial forms of FVH have been reported, albeit with intervals of years between successive cases [20–22]. These observations suggest that FVH may result from monogenic inborn errors of liver immunity to viruses, with penetrance more often incomplete than complete. Severe viral diseases other than FVH can result from such single-gene inborn errors of immunity [23–37]. We recently reported autosomal recessive (AR) IL-18BP deficiency as the first monogenic cause of FVH due to HAV in an otherwise healthy patient [38]. IL-18BP is an antagonist of the cytokine IL-18 [39, 40], which activates the cytotoxicity and IFN-γ production of NK and T cells [41, 42]. The lack of IL-18BP leads to an unleashing of IL-18 activity, resulting in uncontrolled cytotoxicity, killing both HAV-infected and other hepatocytes [38]. We describe here another multiplex family, in which the index patient, with a mild form of early-onset inflammatory bowel disease (EOIBD), died from fulminant hepatitis, and her sister, with a more severe form of EOIBD, developed severe hepatitis due to HAV and died from a combination of these two conditions.

## Results

### Identification of a Homozygous Missense Mutation of *IL10RB*

**Fig. 1:**
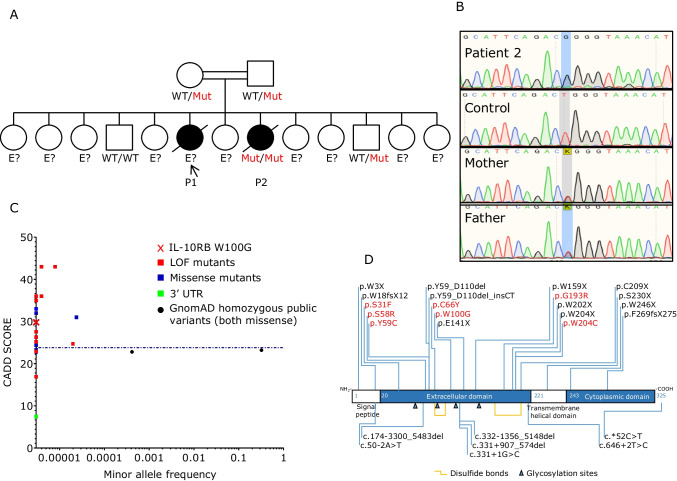
Homozygous tryptophan-to-glycine replacement at position 100 (W100G) of IL-10RB. **A** Pedigree of the family. The patients are shown in black, whereas healthy individuals are shown in white. IL-10RB W100G status is indicated in red, when available. **B** Confirmation by Sanger sequencing of the mutation and its homozygous state in the patient and in the heterozygous state in the parents. **C** Graph showing the CADD 1.3 scores of the published pathogenic IL-10RB variants—where possible—versus their minor allele frequency in gnomAD. W100G IL-10RB is indicated by a red cross, published loss-of-function (LOF, including stop-gain, indel-frameshift, and essential splicing mutations) variants are indicated by red squares, missense mutants are shown as blue squares, and a 3′UTR mutant is indicated by a green square; public homozygous variants—both missense—are indicated by black dots. The 99% mutation significance cutoff (MSC) score is indicated by a dashed line. **D** Schematic diagram of the IL-10RB protein, with the pathogenic mutations marked (missense mutations are shown in red)

We studied a girl who presented a mild form of EOIBD and died from fulminant hepatitis at 6 years of age (P1, Fig. 1A). Her sister, P2, developed a more severe form of infantile IBD at the age of 1 year. She also had an episode of pneumonia of unknown microbial etiology at 2 months of age and suppurative otitis media at 4 months of age, due to *Pseudomonas aeruginosa*. She was treated with intravenous antibiotics and cleared both infections. At 2 years of age, she was hospitalized for lethargy, jaundice, and high liver enzyme levels in a context of HAV infection. She recovered from HAV infection, but her IBD worsened, with multiple perforations of the colon. Total colectomy and ileostomy were performed at the age of 3 years. P2 died suddenly, at the age of 3 years, from multiple organ failure, possibly due to septic shock. The two siblings were born to first-cousin parents originating from and living in Saudi Arabia. Eleven other siblings were healthy. We performed whole exome sequencing on P2, and principal component analysis (PCA) confirmed her Saudi ancestry and parental consanguinity, with a homozygosity rate of 4.71% [43]. No DNA was available for P1. We hypothesized that the genetic disorder in this family would display autosomal recessive (AR) inheritance. We thus selected very rare homozygous non-synonymous variants with a minor allele frequency < 0.001 and prioritized them according to their predicted deleteriousness and potential to impair liver immunity. We identified 18 homozygous candidate variants (Supplementary table 1). The homozygous W100G variant of the *IL10RB* gene, with a CADD score of 29.8, above the 99% mutation significance cutoff (MSC) score of 23.8, was considered to be the best candidate, as it had the highest CADD score and had previously been found in another patient with EOIBD (Fig. 1B) [44]. Its familial segregation was consistent with an AR trait, as parents and three healthy siblings were either heterozygous or WT/WT (Fig. 1A and B). We have no genetic material for any of the remaining siblings. Moreover, there are no homozygous *IL10RB* variants predicted to be LOF in the general population with a CADD score higher than the MSC (Fig. 1C). *IL10RB* encodes the second chain of the receptors for IL-10 (in association with IL-10RA), IL-22 (in association with IL-22RA1), IL-26 (in association with IL-20RA), and IFN-λ (in association with IFNLR1) [45, 46]. Finally, biallelic mutations of *IL10*, *IL10RA*, and *IL10RB* have previously been reported to cause EOIBD [47, 48]. Most of the reported LOF variants of *IL10RB* have a CADD score above the MSC and are private or have a frequency below 10^−4^ (Fig. 1C; Supplementary table 2). The W100G variation has itself been reported in one other patient [44]. Collectively, these data strongly suggest that homozygosity for the W100G variant of *IL10RB* was the cause of EOIBD, and perhaps also of severe hepatitis, in the two affected siblings from this family.

### Impaired Production and Glycosylation of the W100G Protein

**Fig. 2:**
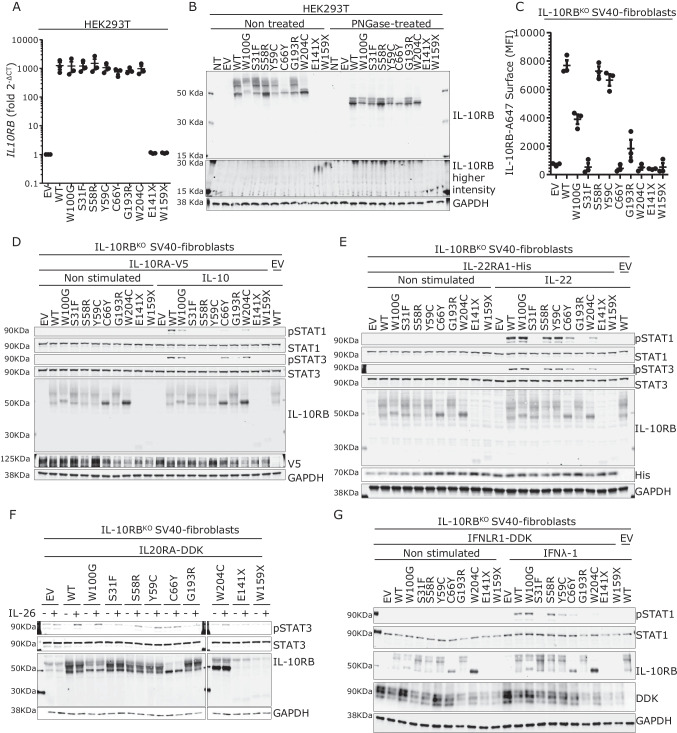
W100G IL-10RB allele characterization and functional assessment in response to IL-10, IL-22, IL-26, and IL-29 stimulation. **A** RT-qPCR results from HEK293T cells transfected for 24 h with the indicated IL-10RB alleles. IL-10RB mRNA levels are normalized against the EV, set as 1. The housekeeping gene *HPRT1* was used as an expression control. The values shown are the means of three independent experiments performed in duplicate ± SEM. **B** Representative western blot showing the level of expression of IL-10RB alleles in HEK293T cells 24 h after transfection, with and without PNGase treatment. **C** Flow cytometry of surface IL-10RB expression on IL-10RB^KO^ SV-40 fibroblasts 24 h after transfection with the indicated IL-10RB alleles. Mean fluorescence intensity (MFI) on the IL-10RB^+^ gate ± SEM is shown. Results from three independent experiments are shown. **D** Response to stimulation with 40 ng/ml IL-10 for 30 min in IL-10RB^KO^ SV-40 fibroblasts transfected with the indicated IL10RB alleles and IL10RA-V5, on a representative immunoblot image for the analysis of STAT1 (pSTAT1) and STAT3 (pSTAT3) phosphorylation. STAT1, STAT3, GAPDH, V5 (IL-10RA), and IL-10RB levels were also assessed. **E** Western blot depicting phospho-STAT1 (pSTAT1) and phospho-STAT3 (pSTAT3) levels in IL-10RB^KO^ SV-40 fibroblasts transfected with the indicated IL-10RB alleles and IL-22RA1-His, after stimulation with 100 ng/ml IL-22 for 30 min. GAPDH was used as a loading control, STAT1, STAT3, His (IL-22RA1), and IL-10RB levels are also shown. **F** Western blot of IL-10RB^KO^ SV40-fibroblasts transfected with the indicated IL-10RB alleles and WT IL-20RA-DDK, after treatment with 100 ng/ml IL-26 for 45 min. The membrane was probed with an antibody specific for phosphorylated STAT3 (pSTAT3). An antibody against GAPDH was used as a loading control; STAT3 and IL-10RB levels are also shown. **G** Western blot showing the detection of phospho-STAT1 (pSTAT1) in response to stimulation with 100 ng/ml IFN-λ1 for 30 min in IL10RB^KO^ SV-40 fibroblasts transfected with the indicated IL-10RB alleles and IFNLR1-DDK. GAPDH was used as a loading control. We also show STAT1, DDK (IFNLR1), and IL-10RB. EV empty vector, WT wild type, SEM standard error of the mean, and p phosphorylated. All western blots were performed at least three times, with the exception of 2F, which was performed twice

None of the previously reported IL-10RB-deficient patients had FVH, and none had been reported to have suffered HAV infection [44, 45, 48–55]. One patient homozygous for W159X developed autoimmune hepatitis [48]. We performed overexpression experiments for the molecular characterization of all reported pathogenic missense *IL10RB* alleles (Table 1) and two stop-gain alleles (E141X and W159X). All these variants had previously been reported to cause EOIBD [44, 47–50, 52, 56–58]. The cytokine response pathway involved was assumed to be that of IL-10 because patients with deficiencies of IL-10 or IL-10RA also present EOIBD. However, the impact of pathogenic *IL10RB* variants on IL-10RB-dependent cytokine response pathways has not been studied experimentally. Predicted loss-of-function (pLOF) variants of *IL10RB* have been reported to cause a premature termination of translation in various domains of the protein, whereas missense pathogenic mutations are clustered in the extracellular domain [44, 48, 50, 52, 56–58] (Fig. 1D). In overexpression experiments in HEK293T cells, all missense variants tested produce amounts of mRNA similar to that for WT *IL10RB*, whereas the two pLOF variants tested (E141X and W159X) produced much less mRNA, suggesting possible degradation by nonsense-mediated mRNA decay (Fig. 2A). At the protein level, WT IL-10RB yielded two major bands, with molecular weights (MW) of about 50 and 60 kDa, suggestive of posttranslational modifications. As expected, both the E141X and W159X proteins had a lower MW and lower level of expression than the WT protein. Four missense proteins, including W100G, were produced in smaller amounts than the WT protein (Fig. 2B). Interestingly, W100G had a higher MW than the WT, whereas C66Y and W204C yielded only the smaller band seen with the WT, suggesting that the pattern of glycosylation might depend on the mutation (Fig. 2B). Following PNGase F treatment, which removes all N-linked oligosaccharides, the two major bands initially seen with the WT and all missense proteins were converted into a triplet, with a major band around 45 kDa, except for C66Y and W204C, for which only the lower band was observed. The MW of W100G was normalized relative to the WT protein, confirming that its initial higher MW was due to aberrant glycosylation. In both the presence and absence of PNGase F treatment, the C66Y and W204C proteins yielded singlet bands, whereas the WT protein yielded a triplet, suggesting that these two variants also alter other posttranslational modifications.

**Table 1:** IL10RB patients carrying at least one missense mutation

Mutation	Number of patients	Molecular mechanism	Patient’s country of origin	Onset of disease (days)	References
S31F	1	Homozygous	Iran	60	Yazdani 2019
S58R	1	Compound heterozygous	USA	< 30	Shouval 2014, 2016, 2017
Y59C	1	Homozygous	Italy	14	Neven 2013, Pigneur 2013
C66Y	1	Homozygous	Bangladesh	45	Kotlartz 2012
W100G	2	Homozygous	France/Saudi Arabia	84/?	Pigneur 2013/this article
G193R	1	Homozygous	Turkey	10	Engelhardt 2013
W204C	2	Compound heterozygous /homozygous	France/China	14/?	Neven 2013, Pigneur 2013, Gong 2019

### Impaired Expression of the W100G Protein on the Cell Surface

We used SV40-transformed fibroblasts from a patient with complete IL-10RB deficiency (IL-10RB^KO^) [47]. In overexpression experiments and experiments with an antibody specific for IL-10RB, we showed that four missense proteins (W100G, S58R, Y59C, and G193R) were expressed at the cell surface, although the median fluorescence intensity (MFI) of two of these proteins (W100G and G193R) was lower than that of the WT protein (Fig. 2C). By contrast, the other three missense proteins tested (S31F, C66Y, W204C) and the two proteins potentially encoded by pLOF variants (E141X and W159X) were undetectable at the cell surface (Fig. 2C). In the same experiments, we also used antibodies against IL-10RB or a tag (Cter-Myc) to analyze intracellular expression. The anti-Myc antibody revealed a normal intracellular expression pattern for all missense proteins and lower levels of the proteins encoded by the two pLOF variants than for the WT protein (Supplementary Fig. 1A). In addition, the antibody against IL-10RB revealed a normal cytosolic expression pattern for S58R, Y59C, and G193R, a slightly lower MFI for W100G, S31F, and C66Y, and no detectable expression of W204C, E141X, and W159X, suggesting that these last three mutant proteins were not recognized by the anti-IL-10RB antibody. As it was not possible to evaluate the surface expression of W204C with the anti-IL-10RB clone at our disposal, we created N-terminally myc-tagged plasmids based on the same backbone. It was unclear whether the IL-10RB epitope recognized by the antibody clone used in our previous experiments was masked on the cell surface by an altered tertiary structure due to the S31F and C66Y mutations. We also, therefore, generated new N-terminally myc-tagged constructs for S31F and C66Y. Following the transfection of cells IL-10RBKO SV-40 fibroblasts for 24 h and surface staining for myc, we observed that the level of surface expression was higher for S31F than for WT IL-10RB, whereas C66Y and W204C had lower levels of expression that nevertheless remained higher than those on EV-transfected cells (Supplementary Fig. 1B). The S31F, C66Y, and W204C protein variants may undergo posttranslational modifications that mask the epitope recognized by the IL-10RB antibody after reaching the surface of the membrane. By contrast, both the W100G and G193R variants were recognized by the antibody against IL-10RB, but their surface expression was weaker than that of WT IL-10RB, perhaps due to cytosolic retention or enhanced receptor degradation. We concluded that the *IL10RB* W100G variant results in lower total protein levels, changes in glycosylation pattern, and lower levels of surface expression. Interestingly, W100G is the only known pathogenic *IL10RB* variant encoding a protein with a MW higher than that of the WT protein.

### Impaired Response to IL-10 for All Missense Variants

In association with IL10RA, IL22RA1, IL20R, and IFNLR1, IL-10RB is involved in signaling downstream from IL-10, IL-22, IL-26, and IFN-λ, respectively [46, 59–63]. The function of the pathogenic IL-10RB proteins, in terms of their response to IL-10RB-dependent cytokines, has not been reported. We investigated whether all variants had the same impact, by transiently transfecting IL-10RB^KO^ SV40-fibroblasts with WT or mutant *IL10RB* (W100G, S31F, S58R, Y59C, C66Y, G193R, W204C, E141E, or W159X), together with WT *IL10RA*. Similar alterations to the glycosylation profiles of the W100G, C66Y, and W204C proteins were observed in this system and in HEK293T cells (Fig. 2D). Following stimulation with IL-10, an impairment of the phosphorylation of STAT1 and STAT3 was observed for W100G, C66Y, and W204C IL-10RB (Fig. 2D). All the other variants (S31F, S58R, Y59Y, G193R, E141X, and W159X) were loss-of-function for the phosphorylation of both STAT1 and STAT3. We also analyzed the induction of CXCL9 induction downstream from STAT1 phosphorylation [64]. Only the WT *IL10RB* allele induced CXCL9 reproducibly in response to IL-10 stimulation (Supplementary Fig. 2A). We found that the *IL10RB* variants S31F, S58R, Y59Y, G193R, E141X, and W159X abolished all responses to IL-10, whereas W100G, C66Y, and W204C impaired the response to IL-10 without abolishing it. These data suggest that the various missense variants had different impacts on cellular responses to IL-10, despite their apparently similar clinical impacts, in terms of EOIBD.

### Impaired or Normal Responses to IL-22, IL-26, and IFN-λ1 Activation in Overexpression Conditions

We then studied cellular responses to IL-22, IL-26, and IFN-λ1 by cotransfecting IL10RB^KO^ cells with WT or mutant *IL10RB,* together with *IL22RA*, *IL20RA*, or *IFNLR*. IL-10RB W100G, S58R, and Y59C responded normally to IL-22 stimulation, in terms of STAT1 and STAT3 phosphorylation (Fig. 2E). The IL-10RB C66Y and W204C proteins responded poorly, and the S31F, G193R, E141X, and W159X proteins did not respond at all. CXCL9 induction mirrored the results for STAT1 phosphorylation (Supplementary Fig. 2B). Following IL-26 stimulation, IL-10RB W100G, S58R, and Y59C responded like the WT protein for STAT3 phosphorylation, whereas S31F, C66Y, G193R, and W204C responded less strongly than the WT protein, and the response was abolished for the E141X and W159X proteins (Fig. 2F). Following IFN-λ1 stimulation, S58R and W100G responded normally, Y59C, C66Y, and W204C responded poorly, and none of the other mutant proteins responded at all, in terms of STAT1 phosphorylation (Fig. 2G) and CXCL9 (Supplementary Fig. 2C). The variant present in the family studied here (W100G) behaved normally, in terms of responses to IL-22, IL-26, and IFN-λ1 in this experimental system. The other variants tested in this in vitro system had responses ranging from normal to abolished, suggesting that IL-22, IL-26, and IFN-λ1 are not crucial for the EOIBD phenotype, although we cannot rule out the possibility that they act as modifiers of this phenotype while underlying other, as yet unknown clinical manifestations. This is consistent with IL-10- and IL-10RA-deficient patients suffering from clinical manifestations of EOIBD that do not seem to be milder than those of IL-10RB-deficient patients [47, 48].

### Impaired Responses to IL-10, IL-22, IL-26, and IFN-λ1 in Cells from a Patient

**Fig. 3:**
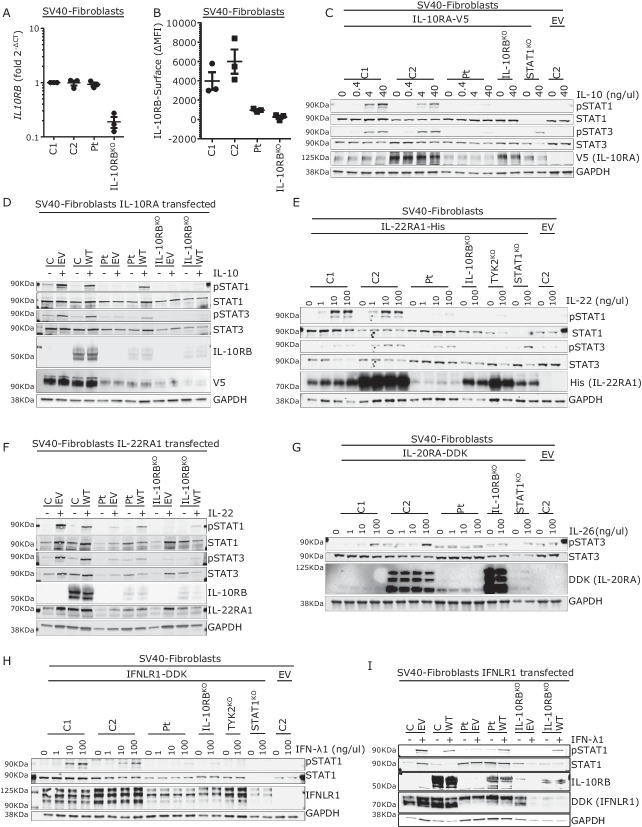
Analysis of patient cells. **A** IL10RB mRNA levels, as determined by RT-qPCR, in patient and control cells. The graph shows the relative expression of IL-10RB mRNA corrected for endogenous levels of HPRT1 mRNA and relative to the first control, set to 1 ± SEM. The values shown are the means of three independent experiments performed in duplicate. **B** The cell surface expression of IL-10RB was assessed by flow cytometry on SV-40 fibroblasts from healthy controls, the patient, and an IL-10RB-deficient patient (IL-10RB^KO^). The results are expressed as the mean fluorescence intensity (MFI) minus the MFI of the isotype control for each (ΔMFI) ± SEM. **C** Response to IL-10 stimulation at the indicated concentrations for 30 min in SV40-fibroblasts from the controls, the patient, and in STAT1^KO^ and IL-10RB^KO^ SV-40 fibroblasts stably transduced with IL-10RA-V5 when indicated, on a representative immunoblot analysis of STAT1 (pSTAT1) and STAT3 (pSTAT3) phosphorylation. We also assessed STAT1, STAT3, GAPDH, and V5 (IL-10RA) levels. **D** Western blot depicting phospho-STAT1 (pSTAT1) and phospho-STAT3 (pSTAT3) levels reconstituted with WT IL-10RB or EV: patient, IL-10RB^KO^, and control SV-40 fibroblasts, after stimulation with 40 ng/ml IL-10 for 30 min. GAPDH was used as a loading control, and STAT1, STAT3, and V5 (IL-10RA1) levels are also shown. **E** Western blot of control, patient, STAT1^KO^, TYK2^KO^, and IL-10RB^KO^ SV40-fibroblasts stably transduced with WT IL-22RA1-His when indicated, after treatment with IL-22 at the indicated concentrations for 30 min. The membrane was probed with antibodies specific for phosphorylated STAT1 (pSTAT1) and phosphorylated STAT3 (pSTAT3). An antibody against GAPDH was used as a loading control; STAT1, STAT3, His (IL-22RA1), and IL-10RB levels are also shown. **F** Western blot showing the detection of phospho-STAT1 (pSTAT1) and phospho-STAT3 (pSTAT3) in response to stimulation with 10 ng/ml IL-22 for 30 min, in control, patient, and IL-10RB^KO^ SV-40 fibroblasts stably transduced with IL-22RA1-His and transiently transfected with WT IL-10RB or EV. GAPDH was used as a loading control. We also show STAT1, STAT3, IL-10RB, and His (IL-22RA1). **G** Response to IL-26 stimulation at the indicated concentrations for 45 min in control, patient, and IL-10RB^KO^ SV-40 fibroblasts stably transduced with IL-20RA-DDK when indicated, on a representative immunoblot analyzing STAT3 (pSTAT3) phosphorylation. STAT3, GAPDH, and DDK (IL-20RA) levels were also assessed. **H** Western blot of control, patient, STAT1^KO^, TYK2^KO^, and IL-10RB^KO^ SV40-fibroblasts stably transduced with WT IFNLR1-DDK when indicated, after treatment with IFN-λ1 at the indicated concentrations for 30 min. The membrane was probed with an antibody specific for phosphorylated STAT1 (pSTAT1). An antibody against GAPDH was used as a loading control; STAT1, IFNLR1, and IL-10RB levels are also shown. **I** Western blot showing the detection of phospho-STAT1 (pSTAT1) in response to stimulation with 100 ng/ml IFN-λ1 for 30 min in control, patient, and IL-10RB^KO^ SV-40 fibroblasts stably transduced with IL-22RA1-His and transiently transfected with WT IL-10RB or EV. GAPDH was used as a loading control. We also show STAT1, IL-10RB, and DDK (IFNLR1). All western blots were performed at least three times, with the exception of G, which was performed twice. C control, Pt patient, WT wild type, SEM standard error of the mean, NT non-transfected, EV empty vector, and p phosphorylated

No cells were available from the two deceased siblings. We, therefore, used fibroblasts from another IL-10RB^W100G/W100G^ patient [44] (referred to here as “patient cells”) for these experiments. We showed that patient cells had normal levels of *IL10RB* mRNAs, as measured by RT-qPCR, whereas the levels of these mRNAs were low in IL-10RB^KO^ cells (Fig. 3A). We investigated the expression of IL-10RB on the cell surface by flow cytometry. We found much lower levels of IL-10RB on the surface of patient and IL-10RB^KO^ cells than on the surface of control cells (Fig. 3B). We then stably transduced patient, IL-10RB^KO^, and control cells separately with each of the four coreceptors of IL-10RB. In response to IL-10 stimulation, patient cells displayed low levels of STAT1 and STAT3 protein phosphorylation and no CXCL9 mRNA induction (Fig. 3C and Supplementary Fig. 3A). STAT1^KO^ cells did not respond to IL-10, in terms of STAT1 phosphorylation or the induction of CXCL9 mRNA. IL-10RB^KO^ cells did not respond to IL-10 stimulation. The transfection of patient cells with WT IL-10RB and IL-10RA rescued the phenotype in terms of STAT1 and STAT3 phosphorylation (Fig. 3D). Cellular responses to IL-22 were also impaired in patient SV40-fibroblasts, with lower levels of STAT1 phosphorylation and CXCL9 induction but with normal STAT3 phosphorylation (Fig. 3E and Supplementary Fig. 3B). The reconstitution of patient cells with WT IL-10RB rescued the IL-22 response defect, as in IL-10RB^KO^ cells transfected with IL-10RA (Fig. 3F). Following IL-26 stimulation, no STAT3 phosphorylation was detected in patient cells (Fig. 3G). We were unable to assess IL-10RB complementation and IL-26 stimulation. The stimulation of patient SV40-fibroblasts with IFN-λ1 resulted in lower levels of STAT1 phosphorylation and CXCL9 mRNA induction than for control cells (Fig. 3I and Supplementary Fig. 3C). In patient cells, IL-10RB reconstitution and IFNLR1 transfection increased STAT1 phosphorylation in response to IFN-λ1 stimulation (Fig. 3I). Overall, functional assays on IL-10RB^W100G/W100G^ patient cells revealed a more profound defect than was observed when the W100G IL-10RB allele was overexpressed in isolation. In the overexpression system, we observed a defect upon IL-10 stimulation, whereas patient cells also displayed defects in response to stimulation with IL-22, IL-26, and IFN-λ1. This discrepancy may reflect differences in the cell surface expression of IL-10RB protein in these two experimental settings (overexpression versus endogenous conditions). The defects of patient cells in response to IL-26 stimulation may also be due to the IL-10RB W100G mutation, but it is not possible to draw firm conclusions as we were unable to develop an assay robust enough to test this hypothesis. Given the complementation of the IL-10, IL-22, and IFN-λ1 responses observed with the WT IL-10RB, we conclude that the cells of the IL-10RB^W100G/W100G^ patient present defects of these pathways due to the homozygous *IL10RB* W100G variant.

### mRNA Levels in Human Liver Cells and the Functional Response of Hepatocytes to IL-10RB Axis Components

**Fig. 4:**
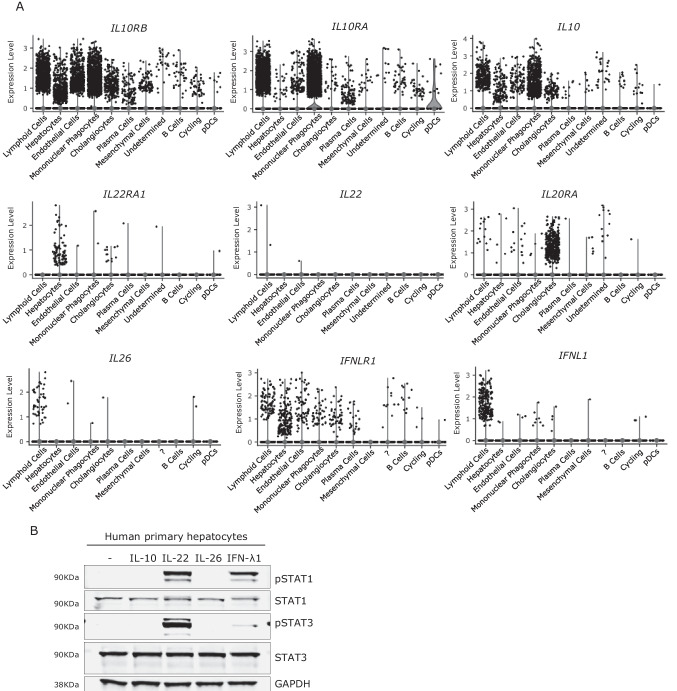
Expression of IL-10RB axis components in the liver*.* **A** Violin plots of single-cell RNA-sequencing data for *IL-10RB*, its coreceptors, and agonists in different liver cell types. Colored violin plots indicate that more than 25% of the cells have an expression level above 0. Cells marked as “?” are cells that it was not possible to identify. **B** Functional validation of the presence or absence of IL-10RB and its coreceptors in human hepatocytes by stimulation with IL-10 (40 ng/ml), IL-22, IL-26, and IFN-λ1 (all at 100 ng/ml) and subsequent phosphorylation of STAT1 and STAT3; one western blot representative of two performed is shown. GAPDH was used as a housekeeping control

We used single-cell RNA-sequencing data for the human liver obtained by integrating publicly available datasets for 28 individuals, to study the abundance of *IL10RB-*related transcripts in the basal state (Fig. 4A) [65]. *IL10RB* mRNA is detected in most of the cell types present in the human liver, including hepatocytes, cholangiocytes, endothelial cells, macrophages/monocytes, and lymphoid cells. By contrast, *IL10RA* mRNA is mostly restricted to macrophages/monocytes, plasmacytoid dendritic cells (pDC), and lymphoid cells, although it is also detected in a small number of other cell types. *IL22RA1* is detected only in a small number of hepatocytes and cholangiocytes. Cholangiocytes are the main source of *IL20RA* mRNA in the liver, whereas the transcript of *IFNLR1*, like that of *IL10RB*, is detected in most liver cell types, but in smaller amounts. The *IL10* transcript is produced mostly in macrophages/monocytes, lymphoid cells, hepatocytes, cholangiocytes, and endothelial cells. *IL22* mRNA is barely detected in the liver, whereas *IL26* mRNA is present in only a small proportion of lymphoid cells. Finally, *IFNL1* mRNA is restricted to lymphoid cells. For validation of these mRNA results, we stimulated previously frozen human hepatocytes with IL-10, IL-22, IL-26, and IFN-λ1 (Fig. 4B). Hepatocytes responded strongly to IL-22 and IFN-λ1 in terms of STAT1 and STAT3 phosphorylation. By contrast, we detected no response to IL-10 or IL-26. These in vitro results are consistent with the mRNA data obtained in vivo for cytokines and their receptors. They also suggest that the acute liver failure may be a consequence of impaired liver-intrinsic immunity, which is not necessarily hepatocyte-intrinsic.

## Discussion

We report AR IL-10RB deficiency as a monogenic cause of FVH. A diagnosis of IL-10RB deficiency should be considered in patients with FVH, particularly, but not exclusively, those with pre-existing infantile or EOIBD. Our findings suggest that the disruption of the IL-10 response pathway may be largely responsible for FVH upon HAV infection, perhaps together with disruption of the IL-22 and/or IFN-λ pathways. Patients with IL-10 or IL-10RA deficiency may therefore be particularly prone to FVH. For the prevention of FVH, patients diagnosed with an IL-10, IL-10RA, or IL-10RB deficiency, whether asymptomatic or with EOIBD, whether before or after hematopoietic stem cell transplantation (HSCT), should be vaccinated against liver-tropic viruses, including HAV and HBV in particular. It is unknown whether FVH has a hematopoietic mechanism in these patients, but the presence of IL10RA mRNA in the liver, mostly in macrophages/monocytes, pDCs, and lymphoid cells, suggests that this could be the case. We previously reported IL-18BP deficiency as the first genetic cause of FVH after HAV infection [38]. IL-18BP is an antagonist of IL-18, which is an inflammatory IFN-γ-inducing cytokine [39, 40, 42]. The lack of IL-18BP unleashes the effects of IL-18, enhancing the cytotoxicity of NK and probably B cells, thereby enhancing the activation of macrophages, probably through excessive IFN-γ production. Strikingly, IL-10 is an anti-inflammatory cytokine, a potent deactivator of phagocytes, and, as such, a natural antidote to IFN-γ [66]. Enhanced and unregulated IFN-γ activity can thus result from the abolition of cellular responses to IL-10. Deficiencies of IL-18BP and IL-10RB result in enhanced inflammation, including enhanced IFN-γ production [38, 67]. It is tempting to speculate that enhanced IFN-γ production is a core mechanism underlying FVH in patients with these two genetic disorders.

Our patients suffered from mild or severe forms of infantile or EOIBD. One presented a failure to thrive, necessitating ileostomy and colostomy. AR IL-10RB deficiency can also underlie perianal lesions, skin folliculitis, B-cell lymphoma, and arthritis, which were not seen in our patients [44, 48, 52, 58, 68]. Not all of the reported patients had B-cell lymphoma, arthritis, or skin folliculitis. By contrast, all the published patients presented with IBD [44, 47–50, 52, 56–58]. These clinical differences do not seem to be related to the causal variant, as different outcomes were reported for patients carrying the same variant [47]. This situation is exemplified by the first family described, in which two affected members were homozygous for the W159X *IL10RB* allele, but one of these patients had more severe clinical manifestations than his sister [47]. Previous studies have focused on functional assays in response to IL-10. The response to IL-22 has been reported in one patient [49]. We also tested cellular responses to IL-26 and IFN-λ1. Our study of the consequences of AR IL-10RB deficiency, in terms of cellular responses to IL-10, IL-22, IL-26, and IFN-λ1, has interesting implications for the pathogenesis of EOIBD. The EOIBD phenotype was known to be caused by the impact of IL-10RB mutations on responses to IL-10, because inherited IL-10 and IL-10RA deficiencies are clinical phenocopies of inherited IL-10RB deficiency, at least in terms of their intestinal phenotypes [47, 48]. We confirm that this is the case, as most mutations impaired IL-10 but not IL-22, IL-26, and IFN-λ1 responses, at least in our overexpression system (Table 2). This is consistent with the EOIBD phenotype being hematological, as patients with IL10RB deficiency undergoing HSCT recover, in terms of intestinal clinical manifestations [48, 69]. Unlike the other three IL-10RB-dependent cytokine and receptor pairs, both IL-10 and its receptor are expressed in leukocytes.

**Table 2:** Summary of results in the overexpression system

Stimulation	Read out	IL-10RB overexpression system									
		WT	W100G	S31F	S58R	Y59C	C66Y	S193R	W204C	E141X	W159X
IL-10	WB-pSTAT1	+ + +	+	-	-	-	+	-	+	-	-
	WB-pSTAT3	+ + +	+ +	-	-	-	+ +	-	+ +	-	-
	qPCR-CXCL9	+ + +	-	-	-	-	-	-	-	-	-
IL-22	WB-pSTAT1	+ + +	+ + +	-	+ + +	+ + +	+	-	+	-	-
	WB-pSTAT3	+ + +	+ + +	-	+ + +	+ + +	+	-	+	-	-
	qPCR-CXCL9	+ + +	+ + +	-	+ + +	+ + +	+	-	+	-	-
IL-26	WB-pSTAT3	+ + +	+ + +	+	+ + +	+ + +	+	+	+ +	-	-
IFN-λ1	WB-pSTAT1	+ + +	+ + +	-	+ + +	+ +	+	-	+	-	-
	qPCR-CXCL9	+ + +	+ + +	-	+ + +	+	+	-	+	-	-

**Fig. 5:**
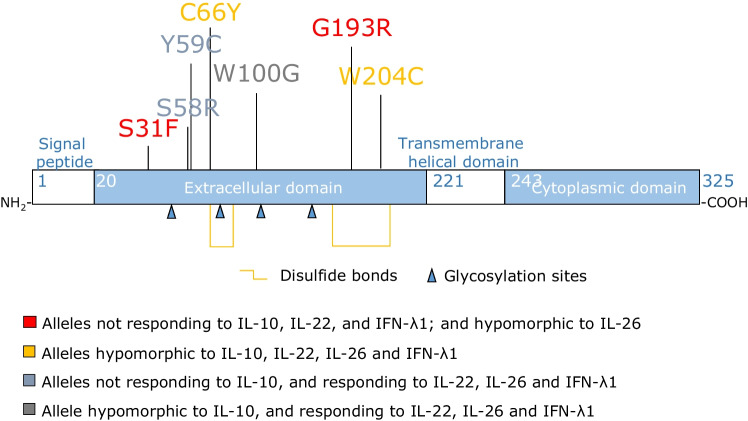
Missense mutations of IL10RB have different cytokine-dependent functional effects. Schematic representation of the IL10RB protein and all the reported missense variants. The code color illustrates the response of all IL10RB-dependent cytokines

Different patterns in the response of the studied missense IL-10RB mutations in overexpression systems are emerging (Fig. 5). Interestingly, disruption of the cellular responses to IL-22, and IFN-λ1 by S31F and G193R mutations, and by stop mutations, does not seem to underlie any particular clinical phenotype, at least until the ages at which death or HSCT occur in the patients [50, 57]. Moreover, IL-10RB C66Y and W204C are both hypomorphic in terms of responses to IL-10, IL-22, IL-26, and IFN-λ1, and they do not give rise to the upper band when overexpressed. These mutations may change the three-dimensional structure of the protein by disrupting or creating disulfide bonds. The IL-10RB S58R and Y59C variants do not respond to IL-10 but do respond to IL-22, IL-26, and IFN-λ1. This finding is consistent with previous studies reporting that the Y59 residue is located in loop 2 of IL-10RB, which is important for IL-10RB/IL-10 interaction [70] but not for IL-10RB/IL-22 interaction. At any rate, IL-10RB W100G is the only reported missense mutation of IL-10RB affecting only the response to IL-10 in our overexpression system in IL-10RB^KO^ cells. Unfortunately, we did not have access to fibroblasts from patients carrying other missense mutations of IL-10RB and were therefore unable to test their response to other cytokines. The different patterns of cellular responses to these four cytokines may underlie or modify the natural history of disease in the patients. Further studies are required to compare the clinical manifestations and cellular responses to IL-10, IL-22, IL-26, and IFN-λ1 of patients with IL-10RB deficiency, with or without fulminant viral hepatitis.

## Methods

### Whole Exome Sequencing (WES) and Sanger Sequencing

DNA was isolated from blood with the iPrep PureLink gDNA Blood Kit and iPrep Instruments, in accordance with the manufacturer’s instructions (Life Technologies). The whole exome sequencing and annotation of the variants were previously described by Belkaya et al. [38]. Variant calls were filtered by removing those with a genotype quality < 50, depth of coverage < 5, no consensus coding sequence, gene damage index > 13.84 [71], allele frequency in any population present in GnomAD > 0.001, CADD score V3.1 > MSC [72], and presence on the blacklist [73]. Only variants annotated as indel-in-frame, indel-frameshift, start-lost, missense, nonsense, stop-lost, and splicing were retained and manually checked in Alamut 2.9.0, to verify that they were actually present.

The IL-10RB W100G mutation was checked by Sanger sequencing with the forward primer TGTCTCATAAATCACATGCCCC and the reverse primer CTTGTGCAAGGCCATCCATTT; for the IL-10RB W159X mutation, forward primer AGCAGTGTACTTCCGTGGAC and reverse primer ACCAGTCCATAAGGTGCTGC were used with *Taq* DNA polymerase (Applied Biosystems, Invitrogen), to amplify the sequence from genomic DNA. The PCR products were analyzed by electrophoresis in a 1% agarose gel. The PCR products were then purified with Sephadex G-50 Superfine resin (GE Healthcare). Sequencing was performed by dideoxynucleotide termination, with the BigDye Terminator kit (Applied Biosystems). Purification was repeated, with Sephadex G-50 Superfine resin, before sequencing on a 3700 apparatus (Applied Biosystems). Results were analyzed with DNA Baser 4.36.0.2 software.

### Cell Culture

SV40-fibroblasts from the published STAT1^c.1928insA/c.1928insA^ (STAT1^KO^)[74], TYK2 (TYK2^KO^), IL10RB^W100G/W100G^ (patient) [43], and IL10RB^W159X/W159X^ (IL-10RB^KO^) patients were used in this study. SV40-transformed fibroblasts (SV40-fibroblasts) were cultured in DMEM supplemented with 10% fetal calf serum (FCS Thermo Fisher Scientific). Cells were cultured at 37 °C, under an atmosphere containing 5% CO_2_.

Human primary hepatocytes were purchased (Cytes Biotechnologies), thawed, and used to seed the media provided by the manufacturer, according to the manufacturer’s instructions: Hepatocyte Thawing Medium, Hepatocyte Plating Medium, and Hepatocyte Maintenance Medium (all from Cytes Biotechnologies). Seeding was performed 24 h before stimulation.

### Plasmids, Transfection, and Transduction

Cells were transfected in the presence of Lipofectamine LTX (Invitrogen), in accordance with the manufacturer’s instructions. The pLX302-IL10RA-V5 plasmid and pLX302 empty vector were purchased from Addgene. pCMV3-IL22RA1-C-His, pCMV3-SP-Myc-IL-10RB, and pCMV3 empty vector were purchased from Sinobiological. All other vectors were purchased from Origene: pCMV6-IL10RB-Myc-DDK, pCMV6-IFNLR1-Myc-DDK (transcript variant 1), pCMV6-IL20RA-Myc-DDK, and pCMV6 empty vector. The mutant alleles for S31F, S58R, Y59C, C66Y, W100G, and G193R were obtained, and the DDK tag on pCMV6-IL10RB-Myc-DDK was eliminated by site-directed mutagenesis (QuikChange II XL; Agilent Technologies), according to the kit manufacturer’s instructions. For the preparation of pCMV6-E141X-IL10RB-Myc and pCMV6-W159X-IL10RB-Myc, we designed reverse primers binding to the plasmid from the stop codons and forward primers binding at the start of the myc tag. We used the CloneAmp HiFi Premix (Takara) for amplification, according to the protocol provided by the manufacturer. We then purified the PCR products with a DNA PCR purification kit (Qiagen), blunted the ends with the Quick Blunting Kit (New England Biolabs), and ligated them with the Quick Ligation Kit (New England Biolabs), for the transformation of competent NEB 10-beta *E. coli* cells (High Efficiency, New England Biolabs) in accordance with the manufacturer’s instructions. All plasmids were inspected by sequencing the full-length ORF, the regions in which it inserted into the backbone and tags. All primers are available upon request.

SV40-fibroblasts were transduced with IL-10RA, IL-20RA, IL-22RA1, and IFNLR1 as described elsewhere [75], with the plasmids mentioned above, with the tags retained. The only difference from the published protocol was the addition of a double purification step for the virus, initially with a filter with 5 nm pores (Millex Merck Millipore) and then with a filter with 0.2 nm pores (Pall Corporation).

For all experiments requiring transient transfection, we used half a million cells to seed the wells of 12-well plates. The following day, the cells were transfected in the presence of the largest recommended amount of Lipofectamine LTX plus (Thermo Fisher Scientific), in accordance with the manufacturer’s instructions. Cells were harvested or stimulated 24 h later.

### Real-Time Quantitative PCRs

Total RNA was extracted with the RNeasy Plus Mini Kit (Qiagen). RNA was reverse-transcribed directly, with the High-Capacity RNA-to-cDNA Master Mix (Applied Biosystems). Q-PCR was performed with the Applied Biosystems Assays-on-Demand probes/primers specific for CXCL9 (Hs00171065_m1) and IL-10RB (Hs00175123_m1). HPRT1 (Hs99999909_m1) was used as an endogenous control for CXCL9 and IL-10RB. The results are expressed according to the ΔΔCt method, as described by the manufacturer.

### Flow Cytometry

Cells were treated with trypsin (Thermo Fisher Scientific) and washed with staining buffer (0.5% FCS in PBS) to prepare them for staining. For the experiments in the overexpression system, IL-10RB Alexa Fluor 647 (BD Mouse IgG1, κ Clone #90,220), Myc-FITC (Miltenyi Biotec), and IL-10RB IL-10RB PE (Bio-Techne, Mouse IgG1 Clone #90,220) antibodies were used in conjunction with the BD Cytofix/Cytoperm kit, according to the manufacturer’s instructions. For patient cells, staining was performed in 100 µl of staining buffer with IL-10RB PE or isotype control (Bio-Techne, Mouse IgG1 Clone #90,220 and mouse IgG1 PE isotype), on ice, for 1 h. The acquisition was performed with a Gallios cytometer (Beckman Coulter), with FlowJo used for analysis.

### PNGase Treatment and Cytokine Stimulation

Cells were treated with PNGase F (New England Biolabs) according to the manufacturer’s instructions. The cells were stimulated with IL-10, IL-22, IL-26, and IFN-λ1, all from R&D Systems, at the time and concentrations indicated in the corresponding figures. In all cases, cells were plated in fresh medium the day before stimulation. All experiments were performed at least three times.

### Immunoblotting

Total protein was extracted from the cells in a lysis buffer containing 1% NP-40 (Fluka), 20 mM Tris–HCl pH 7.4 (Tris MP Biomedicals, HCl Sigma), 140 mM NaCl (VWR), and 2 mM EDTA (MP Biomedicals); supplemented with 100 mM orthovanadate (Sigma), 200 mM PMSF (Sigma), proteinase inhibitor cocktail mix (Roche), phosSTOP (Roche), and 0.1 mM DTT (Invitrogen). Protein fractions were separated by SDS-PAGE and electrotransferred onto nitrocellulose membranes (Biorad). The following primary Abs were used: mouse anti-phosphorylated Y701 STAT1 (BD), mouse anti-STAT1 (BD), rabbit anti-phosphorylated Y705 STAT3 (Cell Signaling Technology), rabbit anti-STAT3 (Cell Signaling Technology), goat anti-IL-10RB (Bio-Techne), mouse HRP-conjugated anti-His (Santa Cruz Biotechnology), mouse anti-V5 (Invitrogen), mouse anti-GAPDH (Santa Cruz Biotechnology) or rabbit anti-GAPDH (Santa Cruz Biotechnology), and mouse HRP-conjugated anti-DDK (Origene). Antibody binding was detected by incubation with HRP-conjugated anti-mouse, anti-goat, or anti-rabbit secondary Abs (GE Healthcare), with the ECL system (Thermo Fisher Scientific). Antibodies were also detected by fluorescence on the Licor system with secondary antibodies conjugated to IRDye800 or IRDye680 anti-mouse, anti-goat, or anti-rabbit antibodies (all from Licor Biosciences Proteomics).

### Single-Cell RNA-Sequencing Analysis

Data were compiled as described in [65]. Briefly, cells of high quality (> 249 transcripts and < 30% mitochondrial expression) were clustered and identified after the integration of five different publicly available single-cell RNA-seq datasets. Violin plots were constructed from the expression levels of *IL10RB, IL10RA, IL10, IL22RA1, IL22, IL20RA, IL26, IFNLR1,* and *IFNL1* and binned by cell type. All data were analyzed with the Seurat (v 3.2.2) library on R (v. 4.0.2).

## Supplementary Information

Below is the link to the electronic supplementary material.

Supplementary file1 (DOCX 301 KB)

## Data Availability

All data are either included in the manuscript or are available upon request.
