# Supplementary material for: Fulminant Viral Hepatitis in Two Siblings with Inherited IL-10RB Deficiency
Source: J Clin Immunol. 2022 Oct 29;43(2):406–20. doi: 10.1007/s10875-022-01376-5 (PMC9892130; doi:10.1007/s10875-022-01376-5)
Supplement: Supplementary file 1 — Supplementary file1 (DOCX 301 KB) [file 10875_2022_1376_MOESM1_ESM.docx]

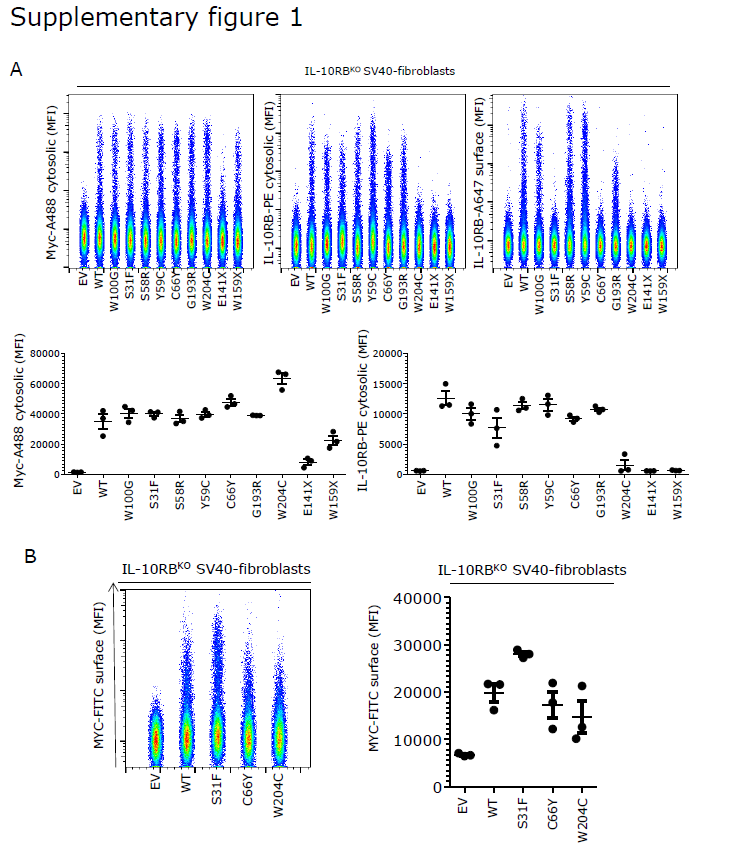


**Supplementary figure 1. W100G IL-10RB surface protein levels. A**. Flow cytometry of the cytosolic tag Myc; and surface and cytosolic IL-10RB expression on transfected IL-10RB^KO^ SV-40 fibroblasts after 24 hours with the indicated IL-10RB-Myc alleles tagged onto the cytosolic C-terminus. We show a dot-plot of one representative experiment for each staining and the accumulated results for three independent experiments for mean fluorescence intensity (MFI) on the IL-10RB^+^ gate ± SEM for cytosolic IL-10RB and Myc. **B**. Flow cytometry of the surface tag Myc; and for transfected IL-10RB^KO^ SV-40 fibroblasts 24 hours after transfection with the indicated IL-10RB-Myc alleles tagged at the N-terminus present at the surface. We show a dot-plot of one representative experiment and the accumulated results for three independent experiments for mean fluorescence intensity (MFI) on the Myc^+^ gate ± SEM for surface Myc.


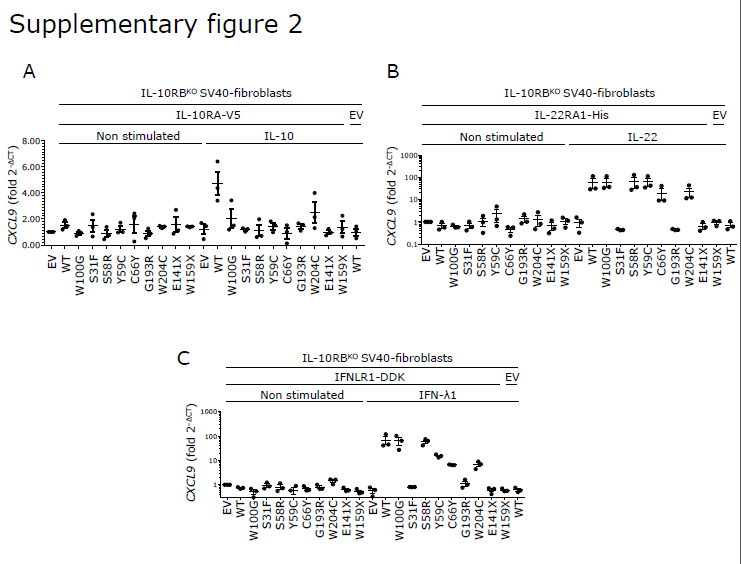


**Supplementary figure 2. Functional assessment of W100G IL-10RB in response to stimulation with IL-10, IL-22 and IFN-λ1. A**. Response to stimulation with 40 ng/ml IL-10 for 2 hours in IL-10RB^KO^ SV-40 fibroblasts transfected with the indicated IL10RB alleles and IL10RA-V5, as assessed by RT-qPCR to determine the levels of CXCL9 mRNA. The housekeeping gene *HPRT1* was used as an expression control. **B**. RT-qPCR results for CXCL9 mRNA levels in IL-10RB^KO^ SV-40 fibroblasts transfected with the indicated IL-10RB alleles and IL-22RA1-His after stimulation with 100 ng/ml IL-22 for 2 hours. The housekeeping gene *HPRT1* was used as an expression control. **C**. Induction of CXCL9 relative to HPRT1, as assessed by RT-qPCR on mRNA extracted from IL-10RB^KO^ SV40-fibroblasts transfected with the indicated IL-10RB alleles and WT IFNLR1-DDK, with or without stimulation for 2 hours with 100 ng/ml IFN-λ1. The values shown are normalized against unstimulated EV, for which the value is set to 1, and are the means of three independent experiments performed in duplicate ± SEM. EV = empty vector, WT = wild type and SEM = standard error of the mean.


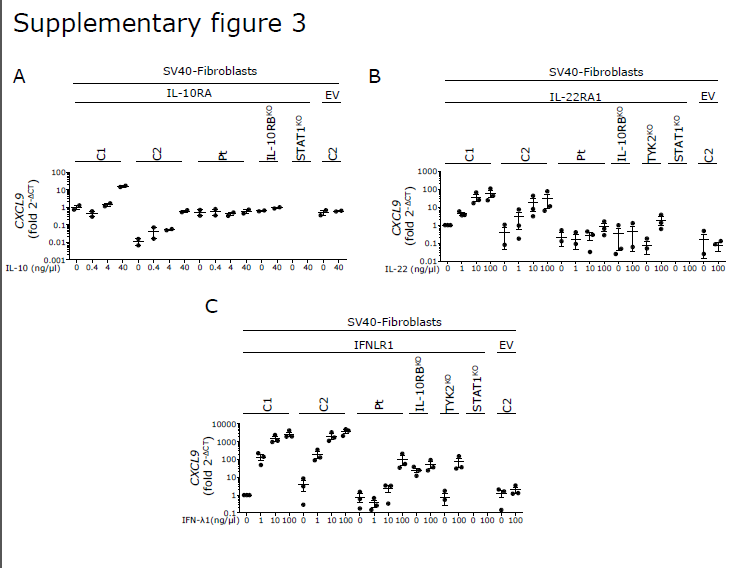


**Supplementary figure 3. RT-qPCR assessment of the function of patient cells in response to stimulation with IL-10, IL-22 and IFN-λ1*.* A**. Response to IL-10 stimulation at the indicated concentrations for 6 hours in control, patient, STAT1^KO^ and IL-10RB^KO^ SV-40 fibroblasts stably transduced with IL-10RA-V5 when indicated. The mRNA levels of CXCL9 were assessed. The housekeeping gene *HPRT1* was used as an expression control. **B**. RT-qPCR of control, patient, STAT1^KO^, TYK2^KO^ and IL-10RB^KO^ SV40-fibroblasts stably transduced with WT IL-22RA1-His when indicated, after treatment with IL-22 at the indicated concentrations for 2 hours. The mRNA levels of CXCL9 were assessed. The housekeeping gene *HPRT1* was used as an expression control. **C**. RT-qPCR of CXCL9 mRNA levels from control, patient, STAT1^KO^, TYK2^KO^ and IL-10RB^KO^ SV40-fibroblasts stably transduced with WT IFNLR1-DDK when indicated, after treatment with IL-29 at the indicated concentrations for 6 hours. The housekeeping gene *HPRT1* was used as an expression control. Results are normalized against the first unstimulated control, for which the value was set to 1. The values shown are the means of three independent experiments performed in duplicate ± SEM, with the exception of panel A, showing one representative experiment of two performed. C = control, Pt = patient, WT = wild type, SEM = standard error of the mean, NT = non-transfected and EV = empty vector. STAT1^KO^ SV40-fibroblast CXCL9 levels were always below the threshold for detection and are, therefore, not marked on the graph.

Supplementary table 1: Homozygous mutations present in patient 2

| Chr | Pos | Ref | Alt | Function | Gene | AAChange | Sift_Pred | Polyphen2  HVAR_Pred | CADD_Phred | MSC | GDI | Known PID? |
| --- | --- | --- | --- | --- | --- | --- | --- | --- | --- | --- | --- | --- |
| 21 | 34649025 | T | G | missense | *IL10RB* | p.Trp100Gly | D,D | D,D,D,D | 29.8 | 23.8 | 10.34 | Yes |
| 2 | 208725979 | T | C | missense | *PLEKHM3* | p.Glu653Gly | D,D | P | 29.7 | 2.31 | 3.26 | No |
| 16 | 88926306 | C | G | missense | *TRAPPC2L* | p.Phe100Leu | D | P,P | 25.9 | 23.84 | 5.19 | No |
| 6 | 41303876 | C | T | missense | *NCR2* | p.Thr35Met | D,D,D | D,D,D | 23.3 | 2.31 | 1.78 | No |
| 20 | 47269910 | C | T | missense | *PREX1* | p.Glu779Lys | T,T | B,B | 20.9 | 2.31 | 13.43 | No |
| 7 | 15725797 | ATGG | A | indel-inframe | *MEOX2* | p.His77del |  |  | 20.5 | 4.78 | 5.44 | No |
| 4 | 8307852 | G | T | missense | *HTRA3* | p.Val451Leu | T | B | 15.35 | 2.31 | 2.85 | No |
| 2 | 238737852 | T | A | splicing | *RBM44* |  |  |  | 10.96 | 2.31 | 4.67 | No |
| 2 | 238737860 | A | G | splicing | *RBM44* | p.Arg868Arg |  |  | 10.79 | 2.31 | 4.67 | No |
| 13 | 38320594 | TA | T | splicing | *TRPC4* |  |  |  | 9.94 | 2.31 | 1.08 | No |
| 2 | 238737854 | T | G | splicing | *RBM44* |  |  |  | 8.267 | 2.31 | 4.67 | No |
| 16 | 12009530 | C | CCCG | indel-inframe | *GSPT1* | p.Gly16dup |  |  | 7.76 | 2.31 | 10.44 | No |
| 7 | 131241029 | G | GGGCGAC | indel-inframe | *PODXL* | p.Ser29_Pro30dup |  |  | 5.726 | 2.31 | 3.35 | No |
| 20 | 32664864 | C | CCAG | indel-inframe | *RALY* | p.Ala230_Gly231insSer |  |  | 4.967 | 2.31 | 5.17 | No |
| 19 | 49657889 | T | TTCC | indel-inframe | *HRC* | p.Glu202dup |  |  | 4.004 | 2.31 | 10.86 | No |
| 13 | 25355965 | GT | G | splicing | *RNF17* |  |  |  | 3.637 | 2.31 | 9.75 | No |
| 3 | 158537415 | CTTTTT | C | splicing | *MFSD1* |  |  |  | 2.493 | 2.31 | 5.61 | No |
| 17 | 78081526 | A | AGCAGCGG | splicing | *GAA* |  |  |  | 0.341 | 0.01 | 7.92 | No |

Supplementary table 2: IL-10RB mutations published with CADD 1.3 score when applicable

| Mutation | Allele frequency | CADD Score | Publication |
| --- | --- | --- | --- |
| p.W3X | Private | 27.6 | Shouval 2014-2017 |
| c.50-2A>T | Private | 16.92 | Shouval 2016-2017 |
| p.W18fsX12 | 0.00001988 | 24.7 | Engelhardt 2013/Shouval 2017 |
| p.S31F | Private | 33 | Yazdani 2019 |
| c.174-3300_5483del | Private | - | Neven 2013/Pigneur 2013 |
| p.S58R | Private | 22.6 | Shouval 2014-2016-2017 |
| p.Y59C | Private | 23.3 | Neven 2013/Pigneur 2013 |
| p.Y59_D110del | Private | - | Shouval 2014-2017 |
| p.Y59_D110del_insCT | Private | - | Charbit-Henrion 2018 |
| p.C66Y | Private | 24.4 | Kotlartz 2012 |
| p.W100G | Private | 29.8 | Pigneur 2013/This article |
| c.331+1G>C | Private | 25.2 | Engelhardt 2013/Shouval 2017/Mukhopadhyay 2020 |
| c.331+907_574del | Private | - | Kotlartz 2012 |
| c.332-1356_5148del | Private | - | Shouval 2014-2017 |
| p.E141X | Private | 36 | Begue 2011/Neven 2013/Pigneur 2013 |
| p.W159X | 0.000003977 | 43 | Glocker 2009/Kotlartz 2012/Shouval 2014-2017/Beser 2015/Karaca 2016 |
| p.G193R | Private | 32 | Engelhardt 2013 |
| p.W202X | Private | - | Shouval 2014-2016-2017 |
| p.W204X | 0.000007953 | 43 | Kotlartz 2012 |
| p.W204C | 0.00002386 | 31 | Neven 2013/Pigneur 2013/Gong 2019 |
| p.C209X | Private | 36 | Petersen 2017 |
| c.646+2T>C | Private | 22.9 | Petersen 2017 |
| p.S230X | Private | 35 | Kotlartz 2012 |
| p.W246X | 0.000003976 | 36 | Huang 2017/Zheng 2019 |
| p.F269fsX275 | Private | 26.3 | Neven 2013/Pigneur 2013 |
| c.*52C>T | Private | 7.461 | Kotlartz 2012/Shouval 2014-2016-2017 |
